# A New High-Performance Liquid Chromatographic Method for the Determination and Distribution of Linalool in *Michelia alba*

**DOI:** 10.3390/molecules15074890

**Published:** 2010-07-12

**Authors:** En-Qin Xia, Yang Song, Xu-Xia Ai, Ya-Jun Guo, Xiang-Rong Xu, Hua-Bin Li

**Affiliations:** 1 Guangdong Provincial Key Laboratory of Food, Nutrition and Health, School of Public Health, Sun Yat-Sen University, Guangzhou 510080, China; E-Mails: enqinxia@163.com (E.X.); sssongyang@163.com (Y.S.); 20040995axx@163.com (X.A.); guoyajunleo@163.com (Y.G.); 2 LMB, South China Sea Institute of Oceanology, Chinese Academy of Sciences, Guangzhou 510301, China; E-Mail: xuxr2000@yahoo.com (X.X.)

**Keywords:** linalool, distribution, high-performance liquid chromatography, *Michelia alba*

## Abstract

A new high-performance liquid chromatographic method with photodiode array detection was established for the determination of linalool in the plant *Michelia alba*. Linalool was extracted from the plant sample with the aid of ultrasound, and was analyzed on a Waters RP C_18_ column (4.6 × 150 mm, 5 μm) using an acetonitrile and water (55:45, v/v) mobile phase at a flow rate of 1.0 mL/min. The column temperature was set at 25 ºC, and the detection wavelength was 210 nm. The linear range of the method was 5–200 μg/mL with a correlation coefficient of 0.9975. The recovery was 92–112%, and the relative standard deviation was 1.85% (n = 9). The present method has been used to study the distribution of linalool in the plant *Michelia alba*. The plant samples include flowers, leaves and tender twigs. Furthermore, leaves included samples in their tender, grown-up and fallen phases, and flowers included samples in their juvenile, middle and whitening phases. The concentrations of linalool in different parts of the plant were 0.21–0.65%, 1.63–4.89% and 0.43% for leaves, flowers and tender twigs, respectively. The results showed that all the plant materials contained relative high concentration of linalool, and juvenile phase flowers contained the highest concentration of linalool. Notably, the fallen leaves also contained high concentrations of linalool, which could be a potential resource of this compound. The results obtained are very helpful for the potential full utilization of this plant.

## 1. Introduction

Linalool (3,7-dimethyl-1,6-octadien-3-ol) is a monoterpene alcohol, which occurs naturally in several aromatic plants [1,2]. Linalool is a much sought after compound in the flavor and fragrance industry, and contributes to the characteristic aroma of a vast number of natural products, such as fruits and spices, as well as tea and chocolate [3,4]. Recent studies showed that linalool has some health-benefits, such as anticancer, anti-inflammatory, antinociceptive, antimicrobial and antioxidant activities as well as mosquito- and sand fly-repelling action [5,6,7,8]. Therefore, the development of a simple and effective method for the determination of linalool in plant samples which could be used to screen or find new natural sources of linalool, and monitor the isolation and purification process of linalool from plant materials is of great importance. 

In the literature, gas chromatography (GC) has been widely used for the determination of organic compounds [9,10,11,12,13], and has also been applied for the determination of linalool in plant samples [14,15,16]. Linalool in plant samples was usually determined by GC-MS [14,15,16], but linalool in extraction solutions that contain an organic solvent and water must be displaced to an organic solvent before injection onto a GC column, and this step could be omitted if the sample were determined by high-performance liquid chromatography (HPLC). In addition, GC-MS was not so widely used as HPLC-UV because GC-MS instruments are much more expensive than HPLC-UV instruments. Furthermore, the determination of the same sample by HPLC-UV often costs less money than for GC-MS. On the other hand, although HPLC has been widely applied for the determination of many substances [17,18,19,20,21,22,23,24,25,26], no report about the determination of linalool in plant samples by HPLC could be found in the literature. Therefore, the aim of this study was to develop a simple, fast and efficient HPLC method for the determination of linalool in the plant *Michelia alba* that is widely planted in South China, and to further investigate the distribution of linalool in different parts of this plant. The results obtained should be useful for the full utilization of this species.

## 2. Results and Discussion 

### 2.1. Optimization of chromatographic condition

**Figure 1:**
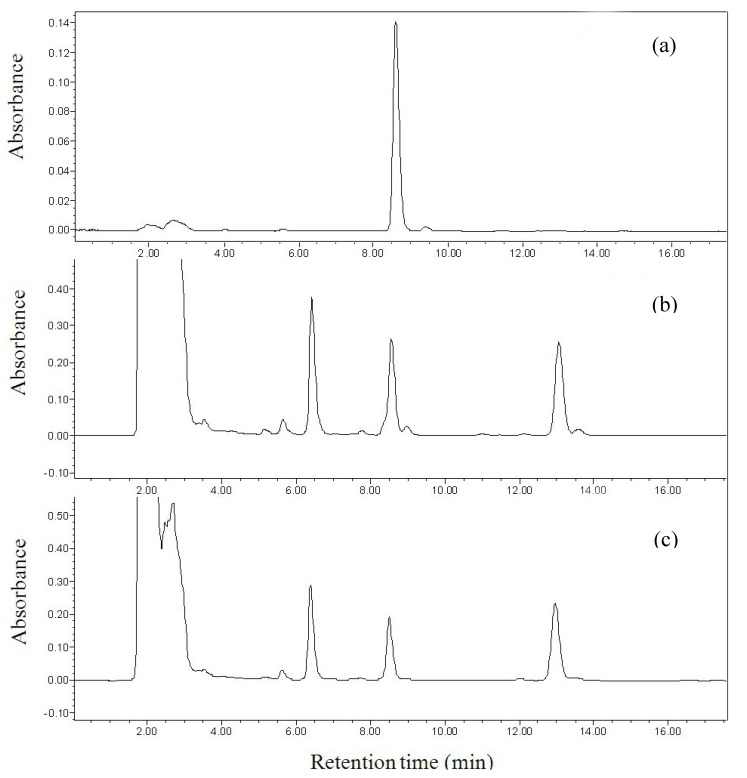
Chromatograms of standard linalool and the extracts *of Michelia alba*: (**a**) standard linalool, (**b**) extract of white flower, and (**c**) extract of tender leaves.

According to the literature [18,19,20], a C_18_ column is often used in reversed-phase HPLC. In this study, a RP C_18_ column (250 mm × 4.6 mm, 5 μm) was tested with different mobile phases. Linalool is an oily compound, and would remain as a neutral molecule during the separation. Thus, the mobile phase may be a simple combination of organic solvent and water, and a buffer solution will not be needed. Methanol and acetonitrile are the most widely used mobile phase components in HPLC separation. However, it was very difficult to separate linalool from other components in the sample when different ratios of methanol-water were used as the mobile phases. In addition, the absorption of methanol was very strong at the detection wavelength of 210 nm. Thus, acetonitrile and deionized water at different ratios were tried as the mobile phases for the separation of linalool from other components in the sample. The separation efficiency was investigated with the acetonitrile composition varying from 50–80% in the mobile phase. The results showed that linalool could be completely separated from other components in the sample with acetonitrile-water (55:45, v/v) as a mobile phase, and the chromatographic peak of linalool was unattached and symmetrical with a retention time of about 8.5 min. Therefore, acetonitrile-water (55:45, v/v) was chosen as the mobile phase in the subsequent experiments. In addition, parameters of the detector were set at 3D mode, and the wavelength was scanned from 190 nm to 700 nm in order to select the optimum detection wavelength. The results showed that the best detection wavelength was 210 nm. Thus, peak area of linalool at 210 nm was used to quantify linalool in the samples throughout this study. Representative chromatograms of a linalool standard and the extracts *of Michelia alba* are shown in Figure 1.

### 2.2. Method validation

The linearity of calibration curve was checked by preparing standard solutions of linalool at seven different concentrations using the stock solution. Each solution was injected in triplicate, and the mean peak area was used. The method showed a good linearity in concentrations ranging from 5 to 200 μg/mL with a correlation coefficient of 0.9975, and the equation is *A* (peak area) = 9296.8 *C* (concentration, μg/mL) - 29250. The limit of detection was 2 μg/mL based on a signal/noise of 3:1 [27]. The precision of method was studied by repeatedly injecting the samples from whitening buds (n = 9), and the relative standard deviation was 1.85%. The recovery of the method was verified using samples with high and low concentrations in triplicate. The recoveries ranged from 92% to 112%, and the relative standard deviations for high and low concentrations were 1.5% and 2.1%, respectively.

### 2.3. Distribution of linalool in the plant Michelia alba

The present method has been used to study the distribution of linalool in the plant *Michelia alba*. Linalool was extracted from the plant samples with ethanol under ultrasonic irradiation, and then analyzed by the proposed method. The samples included different parts of plants, such as flowers, leaves and tender twigs. Furthermore, leaves include different samples in their tender, grown and fallen phases, and flowers in their juvenile, middle and whitening phases were also included. The results are presented in Table 1. From Table 1, the contents of the linalool for tender twigs, leaves and budding flowers were 0.44%, 0.21–0.65%, 1.63–4.89%, respectively. It is apparent from the data that there is a rather large leap between the contents of linalool in leaves and flowers, and levels in twigs and leaves are similar. The content of linalool in juvenile flower buds was near ten times as high as that in leaves and twigs. The results did not agree with those of Mishra *et al*. [28], who reported quite similar levels of concentration of linalool in essential oil of leaves and flowers. The difference might come from different plants studied in each case: *Lippia alba* and *Michelia alba*. It is also possible that the samples in their study might be from blossomed flowers, which had released a lot of aromatic compounds before sampling.

Among different phases of budding flowers, juvenile buds contained the highest concentration of linalool, accounting for 4.89% in fresh samples. With the growth of buds, the concentration of linalool decreased gradually, and whitening buds contained the lowest levels of linalool. This phenomenon can be explained by two aspects. On the one hand, the aromatic compounds could be all present in the flowers when the buds came into being, and then the aromatic compound levels decreased during the growth of buds. On the other hand, the whitening buds indeed displayed a noticeable linalool aroma. 

The content of linalool showed abnormal changes in the different phases of leaves (Table 1). The decreasing order was tender, fallen and growth green leaves, and their concentrations were 0.65%, 0.46%, and 0.21%, respectively. The tender leaves contained the highest concentration of aromatic compounds. The content of linalool decreased gradually with the growth of leaves. The fallen leaves had less weight than the grown leaves due to the volatilization of the water, which resulted in an increased relative content of linalool in fallen leaves. 

It was very important to find that the leaves contain quite high concentrations of linalool (Table 1), which were at the same level as those of the plant *Lippia alba* [1,2,4] (Table 2). Compared with the flowers, leaves constitute very a large part of the total plant, and they are conveniently available without injuring the living tree. Furthermore, the fallen leaves contain the relative high content of linalool, which indicated that fallen leaves are a potential resource of linalool.

## 3. Experimental 

### 3.1. Apparatus

The HPLC system used throughout this study was a Waters (Milford, MA, USA) 1525 Binary HPLC Pump separations module, equipped with a Waters 2996 photodiode array detector. The system also included an auto-injector, and a Waters reversed-phase C_18_ analytical column (4.6 mm × 250 mm, 5 μm). The mobile phase was acetonitrile-water (55:45, v/v) with a flow-rate of 1 mL/min, and the wavelength of detection was set at 210 nm. The mobile phase was degassed with ultrasonic waves before operation. Evaluation and quantification were performed with an Empower chromatography system (Waters). The ultrasound-assisted extraction was carried out in a KQ-600E ultrasonic device (Changzhou Nuoji Instrument Company, China) with an ultrasound power of 600 W, heating power of 800 W, and frequencies of 40 kHz, equipped with digital timer and temperature controller. 

### 3.2. Reagents

Linalool was bought from Sigma (St. Louis, MO, USA). Acetonitrile, methanol and ethanol were HPLC grade and were purchased from Merck (Germany). Deionized water was used throughout the experiments. A stock solution of linalool (1,000 μg/mL) was prepared in methanol and was stored at 4 ºC. The calibration standard solutions (5–200 μg/mL) were prepared from the stock solution by serial dilutions with methanol.

### 3.3. Plant Materials and samples treatment

The leaves, flowers and tender twig of *Michelia alba* were collected in December 2009 from Guangzhou, Guangdong Province, China. The leaves and flowers were ground into small pieces and mixed with an appropriate amount of ethanol. After 30 min, these suspensions were irradiated with ultrasound for 20 min. All samples were filtered through 0.45-μm nylon filters, and then injected onto the HPLC column. The samples of flowers were diluted to three times the initial volume with ethanol before injection. All the experiments were carried out in triplicate, and the results were expressed as mean or mean ± SD (standard deviation).

## 4. Conclusions 

A new high-performance liquid chromatographic method with photodiode array detection was established for the determination of linalool. The linear range of method was 5–200 μg/mL with a correlation coefficient of 0.9975. The recovery was 92–112%, and the relative standard deviation was 1.85% (n = 9). The present method was a simple, fast and efficient method for the determination of linalool in plant samples, and has been used to study the distribution of linalool in the plant *Michelia alba*. The concentrations of linalool in different parts of the plant were 0.21–0.65%, 1.63–4.89% and 0.43% for leaves, flowers and tender twigs, respectively. The results showed that all plant materials contained relative high concentration of linalool, among which juvenile phase flowers contained the highest concentration of linalool. Compared with the flowers, leaves constitute very a large part of the plant mass, and they could be used without injuring the living tree. Furthermore, the fallen leaves contain a relatively high content of linalool, which indicated that fallen leaves are a potential resource of linalool. The results obtained should be very helpful for the full utilization of this plant.

## Figures and Tables

**Table 1:** The content of linalool in the different parts of plant *Michelia alba*.

Parts of the plant	Content of linalool (%, wet weight)
tender leaves	0.65 ± 0.012
grown green leaves	0.21 ± 0.004
fallen leaves	0.46 ± 0.009
juvenile buds of flowers	4.89 ± 0.073
middle buds of flowers	2.86 ± 0.049
whitening buds of flowers	1.63 ± 0.033
tender twig	0.44 ± 0.011

**Table 2:** The contents of linalool (%, weight) in the plant *Lippia alba* [1,2,4]

Parts of the plant	Contents of linalool in plant	References
Leaf of *Lippia alba*	0.13%	[2]
Leaf of *Lippia alba*	0.39–0.52%	[1]
Leaf of *Lippia alba*	0.3–0.8%	[4]
